# FGFR Like1 drives esophageal cancer progression via EMT, PI3K/Akt, and notch signalling: insights from clinical data and next‐generation sequencing analysis

**DOI:** 10.1002/2211-5463.70167

**Published:** 2026-01-02

**Authors:** Aprajita Srivastava, Anoop Saraya, Deepak Gunjan, Rinu Sharma

**Affiliations:** ^1^ University School of Biotechnology, Guru Gobind Singh Indraprastha University New Delhi India; ^2^ Department of Gastroenterology All India Institute of Medical Science New Delhi India

**Keywords:** clonogenic, EMT, esophageal cancer, FGFR, invasion, signalling

## Abstract

FGFRL1, one of the least explored FGFRs, has recently been shown to have implications in cancer, but its functional significance in esophageal cancer (EC) remains largely unexplored. We evaluated FGFRL1 expression in EC patients (*n* = 106) using immunohistochemistry, observing a significant increase in preneoplastic and neoplastic tissues (*P* < 0.001, OR = 73.2; AUC = 0.817). RNAi‐coupled next‐generation sequencing revealed enrichment of EMT, PI3K/Akt, and Notch pathways. Quantitative RT‐PCR validated the downregulation of top cluster and Notch pathway genes (*P* < 0.05). Western blot showed decreased phospho‐GSK3β, phospho‐Akt, and mesenchymal markers. TCGA data supported a positive correlation with EMT. Functional assays demonstrated FGFRL1's oncogenic role, showing G1 phase arrest, and reduced proliferation, migration, and invasion (*P* < 0.001). Our study highlights FGFRL1 as a key driver of EC progression.

## Graphical abstract

Clinical analysis reveals significant dysregulation of FGFRL1 in esophageal cancer (EC) patients. RNAi‐coupled next‐generation sequencing (NGS) and *in vitro* study reveal FGFRL1‐mediated EC progression via EMT, PI3K/Akt, and Notch pathways. Functional assays confirm its role in tumor growth, migration, and invasion. FGFRL1 inhibition leads to G1 arrest and reduced oncogenic traits, highlighting it as a promising therapeutic target for precision treatment in EC.

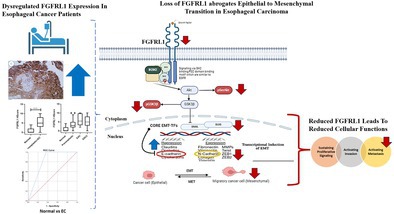

**Abbreviations**
DEGs: differentially expressed genes
EAC: esophageal adenocarcinoma
EC: esophageal cancer
EMT: epithelial‐to‐mesenchymal transition
ESCC: esophageal squamous cell carcinoma
FBS: fetal bovine serum
FGFR: fibroblast growth factor receptor
IHC: immunohistochemical
NGS: next‐generation sequencing
OC: ovarian cancer
OSCC: oral squamous cell carcinoma
SD: standard deviation

Esophageal cancer (EC) ranks eleventh globally in terms of cancer incidence and is associated with a disproportionately high mortality rate, particularly in China and India [1]. Despite advancements in treatment modalities such as chemotherapy, radiotherapy, and surgical resection, the five‐year survival rate for EC remains below 20%. This poor prognosis is largely attributed to delayed diagnosis, the lack of effective biomarkers, and the development of chemoresistance [2]. While targeted therapeutic strategies have shown promise in improving patient outcomes in various cancers, the limited availability of effective therapeutic targets for EC continues to hinder adequate management of this lethal disease. Therefore, there is an urgent need to investigate the key molecular mechanisms driving the malignant progression of esophageal carcinoma, which may lead to the identification of novel therapeutic targets.

The Fibroblast Growth Factor Receptor (FGFR) signalling pathway is one of these key pathways implicated in carcinogenesis. It contributes to cancer progression by acquiring somatic molecular alterations that directly stimulate cancer cell proliferation, survival, and resistance to anticancer agents. In EC, classical FGFR receptors (FGFR1, FGFR2, FGFR3, and FGFR4) are frequently altered through mechanisms such as gene amplification, mutations, alternative splicing, and ligand‐mediated activation [3]. These receptors belong to a family of highly conserved transmembrane tyrosine kinase receptors (RTKs) that regulate critical cellular processes, including proliferation, migration, differentiation, and survival, through downstream pathways such as Ras/Raf–MEK‐MAPKs, PI3K/AKT, PLCγ, and STAT [4].

Recently, a novel member of the FGFR family, Fibroblast Growth Factor Receptor‐Like 1 (FGFRL1), has emerged as a potential target in cancers. FGFRL1 is a transmembrane protein composed of 504 amino acids, and it shares structural similarities with classical FGFRs, including three immunoglobulin‐like (Ig‐like) extracellular domains and a single membrane‐spanning helix. However, FGFRL1 differs from other FGFR family members in its C‐terminal cytoplasmic region, which lacks the classical tyrosine kinase domain responsible for FGFR signalling. Despite this, recent studies have suggested that FGFRL1 may still mediate downstream signalling through specific motifs in its C‐terminal domain. For instance, Zhuang et al. (2010) identified the presence of SH2 domain‐binding motifs, PDZ domain‐binding motifs, ITAM, and ITIM motifs in the C‐terminal region of FGFRL1, which may facilitate intracellular signalling [5]. Furthermore, Silva et al. (2013) demonstrated that an SH2‐binding motif in the short cytoplasmic domain of FGFRL1 interacts with the tyrosine phosphatase SHP1, suggesting that FGFRL1 can enhance ERK1/2 signalling [6].

Emerging evidence highlights the oncogenic potential of FGFRL1 in various cancers. For example, Chen et al. (2020) showed that FGFRL1 positively regulates the expression of ENO1 and activates the PI3K/Akt pathway in small‐cell lung cancer cells [7]. Additionally, reduced expression of Gli1 and Gli2 following FGFRL1 knockdown in ovarian cancer cells suggests a potential role for FGFRL1 in the Hedgehog (Hh) signalling pathway [8]. Moreover, the decrease in phosphorylation of MAPK pathway‐related proteins, such as MEK and ERK, following FGFRL1 knockdown further supports its involvement in tumor progression [9]. Dysregulated expression of FGFRL1 has been reported in several cancers, including ovarian, lung, breast, rectal and prostate cancer, underscoring its potential as a key player in cancer biology [10].

In the context of EC patients, the expression of FGFRL1 and its functional roles and downstream effectors in EC remain poorly understood. This gap in knowledge underscores the need for a thorough investigation of FGFRL1‐mediated signalling in EC. This study aimed to delve deeper into the molecular mechanisms by which FGFRL1 contributes to EC progression. Using RNA interference (RNAi) coupled with next‐generation sequencing (NGS) and overexpression techniques, we seek to unravel the intricate network of interactions and pathways through which FGFRL1 drives the progression of ESCC cells. By elucidating the role of FGFRL1 in key signalling pathways such as EMT, PI3K/Akt, and Notch, this study aimed to provide critical insights into the mechanisms of FGFRL1‐mediated oncogenesis and evaluate its potential as a therapeutic target in EC.

## Materials and methods

### Sample collection

A total of 140 esophageal tissue specimens were collected from patients undergoing endoscopic biopsy at the Department of Gastroenterology, AIIMS. These included 106 tumor biopsies and 34 matched distant nonmalignant tissue samples collected approximately 5 cm away from the tumor site. One portion of each biopsy was fixed in 10% formalin and embedded in paraffin for hematoxylin and eosin (H&E) staining and immunohistochemical (IHC) analysis. The tumor cohort (*n* = 106) was stratified based on histopathological diagnosis into esophageal squamous cell carcinoma (ESCC, *n* = 41), esophageal adenocarcinoma (EAC, *n* = 14), and preneoplastic lesions (*n* = 51). Classification into these categories was based on standard histopathological criteria assessed by trained pathologists. Clinical and pathological data were recorded using a structured proforma that included patient demographics (age, gender), lifestyle factors (tobacco, alcohol, tea consumption, and diet), family history, tumor location (upper, middle, or lower esophagus), and histological differentiation. Written informed consent was obtained from all participants in accordance with institutional ethical guidelines.

### Ethics approval

The study was approved by the institutional research ethics committee of All India Institute of Medical Sciences, New Delhi (reference number: IEC‐545/06.08.2021) and also by the institutional research ethics committee of Guru Gobind Singh Indraprastha University, Dwarka, New Delhi (reference number: GGSIPU/IEC/2021‐A1).

All procedures performed in the studies involving human participants were in accordance with the ethical standards of the Institutional Ethics Committee of All India Institute of Medical Sciences and the Ethics Committee of Guru Gobind Singh Indraprastha University and with the 1964 Helsinki Declaration and its later amendments or comparable ethical standards.

### Immunohistochemistry

Sections (5 μm thick) from paraffin‐embedded blocks of both EC and adjacent nonmalignant tissues were mounted on poly‐L‐lysine‐coated slides. The slides were deparaffinized, rehydrated, and subjected to antigen retrieval using Tris–EDTA buffer. Endogenous peroxidase activity was quenched by incubating sections in 0.3% hydrogen peroxide in methanol for 30 min. Nonspecific binding was minimized by blocking with 1% normal horse serum. The slides were then incubated overnight at 4 °C with rabbit polyclonal anti‐FGFRL1 antibody (1:50 dilution; Cloudclone, Katy, TX, USA). The following day, slides were rinsed and incubated with HRP‐conjugated anti‐rabbit IgG polymer detection reagent (ImmPRESS, Vector Laboratories, Newark, CA, USA) for 30 min at room temperature. Signal detection was performed using diaminobenzidine (DAB) as the chromogen, and nuclei were counterstained with hematoxylin. Immunoreactivity was semiquantitatively assessed using an H‐score system that accounted for both staining intensity and the percentage of positively stained cells. The percentage positivity was graded as: ≤10% = 0, 11–20% = 1, 21–40% = 2, 41–60% = 3, 61–80% = 4, and >81% = 5. Staining intensity was scored as: faint = 1, moderate = 2, and strong = 3. The final H‐score was derived by summing the intensity and distribution scores. To minimize observer bias, two independent reviewers evaluated the slides. In cases of discrepancy between two observers, a joint review was conducted to reach consensus. Based on receiver operating characteristic (ROC) curve analysis, a total H‐score of 2 was set as the threshold for FGFRL1 positivity: Scores below this cut‐off were considered negative (0), and those above were considered positive (1). The information documented included patient demographics (age, gender), lifestyle habits (diet, tea, alcohol, and tobacco use), family history, tumor location (upper, mid, or lower esophagus), and histological differentiation.

### Cell culture and transfection

The human esophageal squamous carcinoma cell line KYSE‐410 (Sigma‐Aldrich (MilliporeSigma, St. Louis, MO, USA, India), UNSPSC Code:41106514) was cultured in RPMI‐1640 medium supplemented with 10% heat‐inactivated fetal bovine serum (FBS) and 1% antibiotics at 37 °C in a humidified atmosphere with 5% CO₂. Transfections were carried out using Lipofectamine 3000 (Invitrogen, Carlsbad, CA, USA) in serum‐ and antibiotic‐free conditions. For knockdown experiments, cells were transfected with 100 nm FGFRL1‐specific siRNA or scrambled control siRNA (Ambion, (Thermo Fisher Scientific, Austin, TX, USA)). For overexpression studies, full‐length human FGFRL1 and a C‐terminal deletion mutant (FGFRL1ΔC) were cloned into the mEGFP‐N1 vector at SalI/BamHI sites, sequence‐verified, and then transfected into KYSE‐410 cells.

### Real‐time polymerase chain reaction

Total RNA was extracted from cell lines (transfected with FGFRL1 siRNA or scrambled sequence siRNA or pCMV‐FGFRL1‐EGFP/pCMV‐FGFRL1ΔC‐EGFP or empty vector) using RNAeasy mini kit (Qiagen, Copenhagen, Denmark) as per the manufacturer's protocol. cDNA was synthesized from 1 μg of total RNA by reverse transcription PCR. Quantitative Real‐Time PCR was conducted in a 25 μL reaction mixture containing 0.5 μm of each gene‐specific primer (Table S1), 12.5 μL SYBR® Premix Ex Taq (Takara Bio, Shiga, Japan), and 2.5 μL of cDNA using a CFX96 Real‐Time PCR system (Bio‐Rad Laboratories, Hercules, CA, USA). PCRs were prepared and heated to 95 °C for 1 min followed by 40 cycles of denaturation at 95 °C for 10 s, annealing at specific Tm for 5 s, and extension at 72 °C for 1 min. Fluorescence detection was performed at the end of each extension step. Relative gene expression was normalized using 5 s RNA as the internal control and calculated using the 2^−ΔΔCT^ method.

### Western blotting

KYSE‐410 cells (transfected with FGFRL1 siRNA or scrambled sequence siRNA or pCMV‐FGFRL1‐EGFP/pCMV‐FGFRL1ΔC‐EGFP or empty vector) were lysed in RIPA buffer (Sigma) and scraped off the dish for total protein isolation. Cells were harvested and total protein extracts were prepared. Briefly, cells were lysed in RIPA buffer and 1x protease inhibitor cocktail. Protein concentration was measured using the Bradford reagent (Sigma), and bovine serum albumin was used as the standard. Proteins were separated by SDS/PAGE and transferred to PVDF membranes by electroblotting. Membranes were blocked with 5% skimmed milk. Primary antibody incubation was done overnight at 4 °C with rabbit polyclonal anti‐FGFRL1 antibody (1:50 dilution; Cloudclone Cat#PAL113Hu01) and polyclonal anti‐GAPDH antibody (house‐keeping protein at 1:600 dilution; Novus Biologicals, LLC, USA, Cat# NB300‐322H), RRID: AB_1660142, PI3K/AKT signalling pathway monoclonal antibodies, namely phospho‐Akt (Ser473, D9E, Cat. #4060), pan‐Akt (C67E7, Cat. #4691), and phospho‐GSK3β (Ser9, D85E12, Cat. #5558) and EMT signalling pathway monoclonal antibodies viz. *N*‐cadherin (D4R1H, Cat. #13116), E‐cadherin (24E10, Cat. #3195), Slug (C19G7, Cat. #9585), Vimentin (D21H3, Cat. #5741), β‐catenin (D10A8, Cat. #8480), ZEB1 (D80D3, Cat. #3396), and Snail (C15D3, Cat. #3879) (1:600 dilution; Cell signalling Technology, MA, USA). Postprimary antibody incubation, membranes were incubated with horseradish peroxidase (HRP)‐conjugated secondary antibodies, and the chemiluminescence of HRP‐conjugated immunolabeled proteins was detected using an enhanced chemiluminescence kit Thermo Scientific (Thermo Fisher, Waltham, MA, USA). The integrated density value (IDV) for each protein was determined using the imagej software and normalized with GAPDH density values.

### mRNA enrichment and library preparation for NGS transcriptome sequencing

500 ng of total RNA was isolated post‐FGFRL1 knockdown or scrambled sequence siRNA. Poly(A) mRNA enrichment was performed using the NEB Next magnetic isolation module (New England Biolabs, Ipswich, MA, USA), followed by library construction using the NEB RNA Library Prep Kit. The quality of libraries was generated using TapeStation Analysis Software 4.1.1 © Agilent Technologies, Inc. 2021. Furthermore, the cDNA was PCR‐amplified and purified, and the final DNA library was eluted in 15 μL of 0.1× TE buffer.

### mRNA NGS data analysis‐bioinformatics and statistics

Paired‐end sequencing was performed on an Illumina HiSeq platform. Data quality was checked using FastQC and MultiQC software. Raw sequence reads were processed to remove adapter sequences and low‐quality bases using fastp. The QC‐passed high‐quality reads were aligned to the GRCh38.p7 human reference genome using HISAT2. Transcript abundance was quantified in FPKM using Cuffdiff. Sample correlations were assessed using Spearman correlation analysis.

### Pathway and functional enrichment analysis

The functional pathway enrichment of the differentially expressed genes (DEGs) was analyzed using the Ingenuity Pathway Analysis software (IPA, QIAGEN, Hilden, North Rhine‐Westphalia, Germany) according to the manufacturer's instructions (Kramer et al., 2014). Pathway identification and molecular functions were accomplished using Fisher's exact test, with Benjamini–Hochberg multiple testing correction. For analysis, DEG expression with (a) log_2_‐fold‐change (log_2_ FC) values between −2 and 2; (b) *P* > 0.05; (c) False Discovery Rate (FDR) > 0.05, and (d) FPKM<1 for all samples were removed. Unrestricted pathway analysis was conducted without metadata alignment. Also, Enricher and Funrich Tools were used to analyze data through the KEGG 2021 Human database, Human Molecular Signatures Database (MSigDB Hallmark) 2020, and Mammalian Phenotype (MP) Ontology (MGI) mammalian phenotype 4, 2021, for profound analysis.

### WST‐1 assay

KYSE‐410 cells were treated with 100 nm FGFRL1siRNA and scrambled sequence siRNA or pCMV‐FGFRL1‐EGFP/pCMV‐FGFRL1ΔC‐EGFP or empty vector, and KYSE‐410 cells were subjected to the WST‐1 proliferation assay at 48 h. Cell Proliferation Reagent WST‐1 (10 μL·well^−1^) was added and incubated for 3 h at 37 °C in 5% CO₂. The plate was shaken thoroughly for 1 min on a shaker. The absorbance of the samples was measured against a background control as a blank using a microplate (ELISA) reader. The wavelength for measuring the absorbance of the product is 450 and 630 nm. Each reading was converted to the percentage of proliferation, which was calculated as follows:

$$\text{Proliferation rate}\left(\%\right)=\begin{array}{l} \left(\mathrm{OD}\;\text{value of the experimental group}\right)/\mathrm{OD}\;\text{value of the control group}\times 100\%. \end{array}$$

### Colony formation assay

KYSE‐410 cells were transfected with FGFRL1 siRNA, scrambled sequence siRNA pCMV‐FGFRL1‐EGFP, pCMV‐FGFRL1ΔC‐EGFP, or empty vector. At 48 h post‐transfection, KYSE‐410 cells were trypsinized and seeded in six‐well plates at a density of 3 × 10^3^ cells·well^−1^. Cells were cultured for 6 days with medium changes every 2 days. Colonies were fixed with prechilled methanol, stained with crystal violet (HiMedia), and imaged. Images of the stained tumor cell colonies were then recorded with a digital camera and colonies containing ≥50 cells were counted using the imagej software.

### Boyden chamber assay

KYSE‐410 cells were transfected with FGFRL1 siRNA or scrambled sequence siRNA. After 48 h, cells were harvested via trypsinization, resuspended in 1× PBS, and seeded at a density of 8 × 10^4^ cells per insert into Boyden chambers—with or without Matrigel coating to assess migration or invasion, respectively. The lower wells contained RPMI supplemented with 10% FBS to serve as a chemoattractant. After 24 h of incubation at 37 °C, noninvading cells were removed from the upper membrane surface. Cells that had migrated or invaded to the lower side were fixed using prechilled methanol, stained, and visualized under an inverted fluorescence microscope for quantification.

### 3D droplet invasion assay

Cells were counted for each drop with a hemocytometer and the required number of cells (50 000/drop) was added into a 1.5‐mL Eppendorf tube in 10% RPMI media for the cells. The tubes were centrifuged at 1000 ***g*** for 5 min at 4 °C. The supernatant media were removed with gentle pipetting. Matrigel was added to the cell pellet in an Eppendorf tube and 10 μL of Matrigel was used per drop and these steps were performed on ice. The Matrigel‐cell droplets were incubated at 37 °C with 5% CO₂ for 20 min to allow polymerization. Once solidified, 2 mL of RPMI supplemented with 10% FBS was added to each well. Cells were imaged every day and the migrating edge outside the Matrigel drop was measured with imagej. A line was drawn with the freehand tool of imagej to measure the entire area with the Matrigel drop and the migrated cells.

### Cell cycle analysis

For cell cycle analysis, cells were washed with PBS, detached using 300 μL of trypsin, and incubated at 37 °C for 1 min. The reaction was neutralized with 700 μL of complete medium, and cells were centrifuged at 3000 rpm for 6 min. Traces of PBS were removed, and then, the pellet was resuspended in 300 μL of PBS to make a single‐cell suspension. Then, chilled 700 μL of 90% methanol was added. The cells were dispersed and the tube was stored at −20 °C overnight. The fixative was discarded and the cell pellet was washed with 1 mL of PBS by centrifuging at 3000 rpm for 6 min. The supernatant was discarded and 1 mL of 100 μg·mL^−1^ RNase A working solution was added. The cell pellet was resuspended and incubated at 37 °C for 30 min. The supernatant was discarded, and 200 μL of propidium iodide solution (50 μg·mL^−1^) was added followed by incubation at 37 °C for 30 min in the dark. After discarding the RNase, 200 μL of propidium iodide solution (50 μg·mL^−1^) was added, and cells were incubated in the dark for 30 min. Excess PI was removed by centrifugation and washing with PBS, and the final cell suspension (500 μL in PBS) was subjected to flow cytometric analysis using a BD LSRFortessa™ X‐20. Data acquisition was analyzed using the flowjo v10.10_CL software.

### Statistical analysis

All experimental data were analyzed using the GraphPad Prism software. Functional assays were performed using at least three independent biological replicates, with each biological replicate containing technical replicates accordingly. Data are presented as mean ± standard deviation (SD). Statistical significance between two groups was assessed using Student's t‐test, while comparisons among multiple groups were evaluated using one‐way ANOVA where appropriate. The Mann–Whitney U test was applied to compare FGFRL1 expression levels between EC tissues and matched distant nonmalignant tissues. Diagnostic performance was assessed using ROC curve analysis, and sensitivity, specificity, and positive predictive value (PPV) were calculated using the following formulas: Sensitivity = TP/(TP + FN), Specificity = TN/(TN + FP), and PPV = TP/(TP + FP). The association between FGFRL1 expression and clinicopathological parameters of ESCC patients was analyzed using the χ^2^ (chi‐square) test. A *P*‐value ≤ 0.05 was considered statistically significant.

### Correlation data retrieval

Correlation analysis was carried out using Correlation R for major EMT markers with FGFRL1 in both EC subtypes, (esophageal squamous cell carcinoma and adenocarcinoma) databases available in the TCGA dataset.

## Results

### Immunohistochemical analysis of FGFRL1 in esophageal cancer patient cohort

**Fig. 1:**
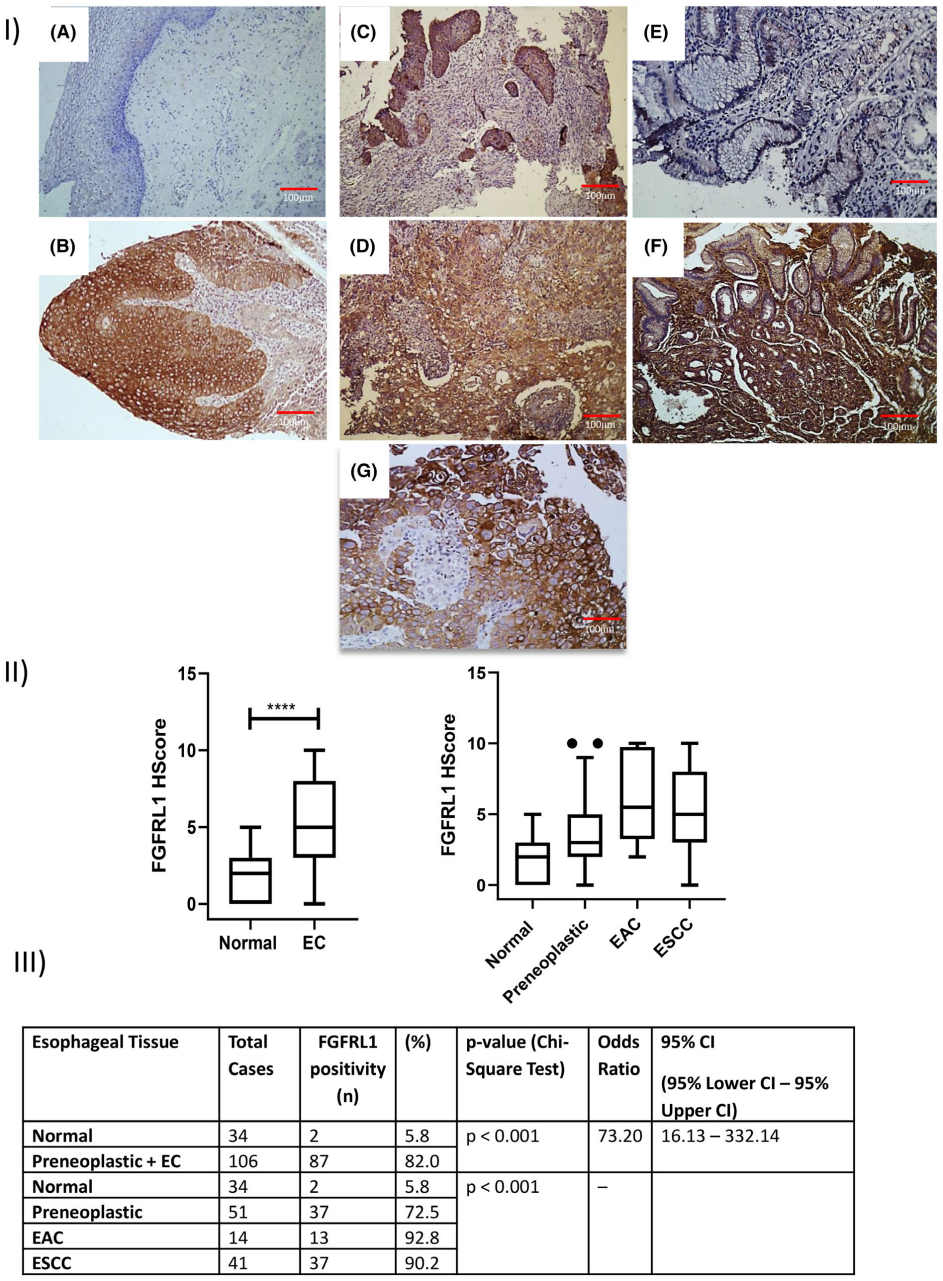
(I) Representative esophageal tissue section immunostained for FGFRL1 in (A) histologically normal tissue (B) dysplastic tissue (C) Moderately differentiated tissue (D) Poorly differentiated tissue (E) Barrett's esophagus & (F) Esophageal adenocarcinoma tissue (Magnification: 10×) (G) Esophageal cancer tissue showing cytoplasmic and membrane staining (Magnification: 20×). (II) (A) Boxplot showing differential expression of FGFRL1 in cancer vs matched distant non‐malignant tissues (*P* < 0.001, Mann–Whitney U test and values are presented as mean ± SD. (B) Boxplot showing expression of FGFRL1 in subtypes of esophageal cancer and normal tissues of EC patients (One‐way ANOVA, *P* < 0.001). (III) Table showing percentage expression of FGFRL1 in subtypes of esophageal cancer and normal tissues of EC patients.

**Fig. 2:**
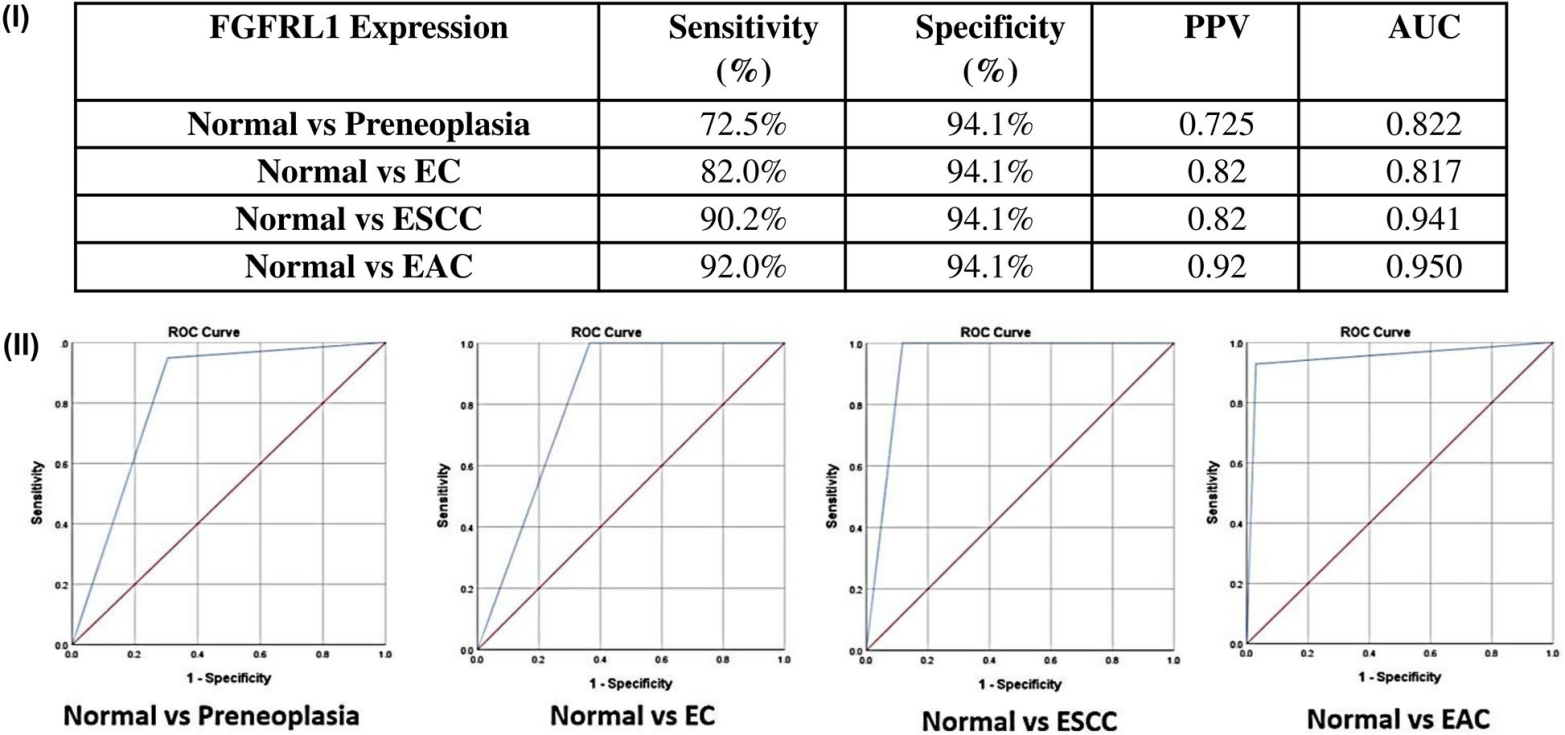
(I) Diagnostic performance of FGFRL1 expression in distinguishing normal esophageal tissues from different pathological conditions. The table summarizes sensitivity, specificity, positive predictive value (PPV), and area under the ROC curve (AUC) for the comparisons: Normal vs. Preneoplasia, Normal vs. Esophageal Cancer (EC), (Normal vs. Esophageal Squamous Cell Carcinoma (ESCC), and Normal vs. Esophageal Adenocarcinoma (EAC). (II) Receiver Operating Characteristic (ROC) curves illustrating the diagnostic performance of FGFRL1 expression in: Normal vs. Preneoplasia, Normal vs. EC, Normal vs. ESCC and Normal vs. EAC. The blue line represents the ROC curve, and the red diagonal line represents the reference line (AUC = 0.5). Higher AUC values indicate better discriminatory ability of FGFRL1 expression in differentiating normal from diseased tissues.

We checked the clinical significance of FGFRL1 protein in EC; its expression was analyzed in clinical specimens from histologically normal (*n*= 34), preneoplastic (*n* = 51), and EAC biopsy samples (*n*= 14) and esophageal squamous cell carcinoma biopsy samples (*n*= 41). The representative images are given in Fig. 1, I. A total of 140 specimens were analyzed, and upregulation of FGFRL1 in 82.0% (87/106) EC tissues as compared to matched distant nonmalignant tissues was found to be statistically significant (*P* < 0.001, OR=73.2). We found significant FGFRL1 positivity within the preneoplastic tissue (72.5%), ESCC (92.8%), and EAC subset (90.2%) as compared to distant nonmalignant tissues (*P* = 0.002) (Fig. 1, II & III). We observed FGFRL1 protein expression in the cytoplasm as well as the membrane of premalignant and EC tissues. No detectable expression of FGFRL1 was observed in the nonmalignant tissue section (Fig. S3). ROC curve analysis yielded an AUC of 0.817 and the highest sensitivity of 82.0% in terms of distinguishing EC tissues from distant nonmalignant tissues and a specificity of 94.1% with a positive predictive value of 0.82 (Fig. 2, I & II). The clinicopathological parameters of EC specimens and their correlation with the expression of FGFRL1 are shown in Table 1. χ^2^ analysis showed no significant association between FGFRL1 expression and site, age, gender, RT, and CT. Only tobacco (*P* = 0.002) showed a significant association with FGFRL1 expression, stating smokers are more likely to have high expression. No significant association was observed between FGFRL1 expression and other habits viz. alcohol drinking, tea consumption, oil consumption, or chili consumption (Table 1).

**Table 1:** Clinicopathological parameters: FGFRL1 positivity in esophageal cancer (EC) cases based on age, gender, treatment, and tumor site. Sociodemographic parameters: FGFRL1 expression in relation to lifestyle factors. Smoking shows a significant association with higher FGFRL1 positivity (*P* = 0.002, OR = 8.10), while other factors show no significant correlation.

Esophageal tissue	Total cases	FGFRL1 positivity, *n* (%)		*P*‐value (Chi‐Square test)	Odds ratio	95% CI (95% lower CI‐95% upper CI)
Clinicopathological parameters						
Preneoplastic + EC	106					
Age						
>40	93	76		0.577	0.810	0.121–2.851
<40	13	11	84.6			
Gender						
Male	63	50	79.0	0.447	0.623	0.217–1.794
Female	43	37	85.4			
Treatment						
Chemotherapy given	1	8	12.5	0.494	0.028	0.0046–0.339
No Chemotherapy given	97	76	78.3			
Radiotherapy given	11	11	76.0	0.205	0.00	0.152–2.621
No Radiotherapy given	94	76	80.8			
Site						
Upper + Mid	89	73	81.4	0.640	0.97	0.234–3.516
Lower	17	14	82.4			
Sociodemographic parameters						
Habits						
Smokers	43	41	95.3	0.002	7.57	1.650–34.795
Non‐Smokers	63	46	73.0			
Alcoholic	26	24	92.3	0.148	3.2	0.695–15.086
Non‐Alcoholic	80	63	78.7			
Tea consumption						
Yes	55	47	85.4	0.446	1.61	0.592–4.408
No	51	40	78.4			
Chillies consumption						
Yes	54	46	85.1	0.454	1.54	0.566–4.207
No	52	41	78.8			
Oil consumption						
Yes	47	41	87.2	0.163	1.93	0.672–5.546
No	59	46	77.9			

### FGFRL1 knockdown affects major signalling pathways in esophageal carcinoma cells

To demonstrate the functional role of FGFRL1 expression in human EC, we silenced FGFRL1 in the esophageal carcinoma cell line (KYSE410) using the RNAi approach. Next, mRNA and protein expression levels post‐FGFRL1 knockdown were recorded.

**Fig. 3:**
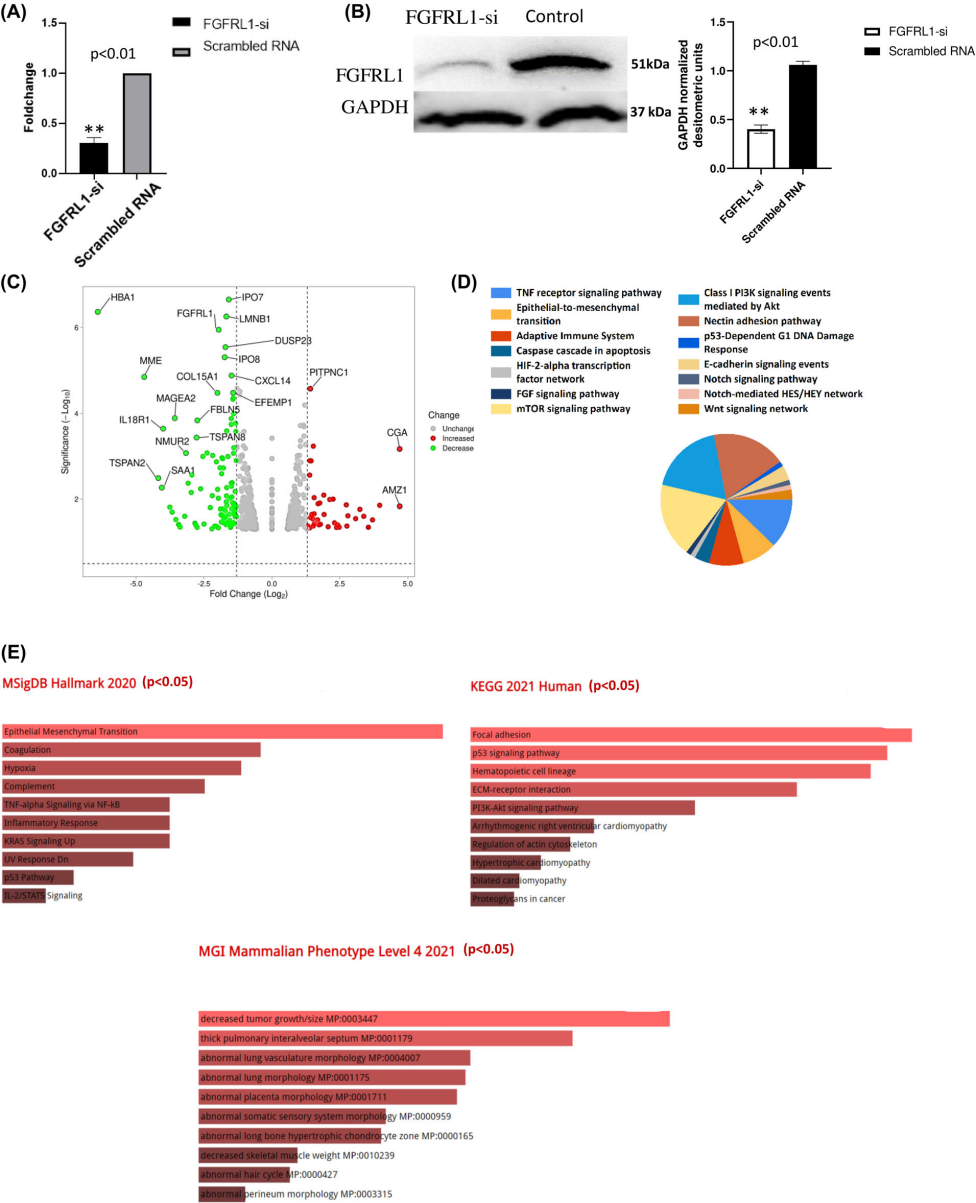
(A) mRNA expression level of FGFRL1 in the KYSE410, 48 post‐FGFRL1knockdown, (B) Protein level expression of FGFRL1 in the KYSE410, 48 h post‐FGFRL1 knockdown. Lane 1, cells transfected with FGFRL1 silencer post 48 h; Lane 2, cells transfected with Scrambled RNA post 48 h (Student's *t*‐test). (C) Volcano plot of DEGs post‐FGFRL1 knockdown KYSE410 and Control. (D, E) Pathway analysis of Downregulated DEGs in NGS profile post‐FGFRL1 knockdown in different databanks using Funrich and Enricher. ** indicates *P* < 0.01 and values are presented as mean ± SD.

Compared with scrambled siRNA‐transfected cells, cells transfected with FGFRL1‐specific siRNA showed a significant decrease of 73% (*P* ≤ 0.01) in mRNA and 71% (*P* ≤ 0.01) in protein expression levels of FGFRL1 post‐48 h (Fig. 3A,B). Next, the changes in the mRNA profile were studied by transcriptome profiling via Illumina Next‐Generation Sequencing. A total of 795 transcripts were recorded as significant DEGs. Furthermore, the DESeq2 software was used to check the differential expression in the FGFRL1 knockdown group, where 121 DEGs were upregulated and 236 were downregulated DEGs. In addition, there were 43 FGFRL1 knockdown group‐specific genes, and 51 were control‐specific genes. The expression profile of differentially expressed genes across the replicates is presented in the volcano plot (Fig. 3C).

The downregulated DEGs and control‐specific genes were analyzed with the Funrich Tool as well as the Enricher Tool which uses the KEGG 2021 Human database, Human Molecular Signatures Database (MSigDB Hallmark) 2020, and Mammalian Phenotype (MP) Ontology (MGI), 2021, (KEGG 2021 database for pathway enrichment) (Fig. 3D,E). Several oncogenic pathways such as Focal adhesion, p53 signalling pathway, and PI3K‐Akt pathway were found as major downregulated signalling networks post‐FGFRL1 knockdown. The MSigDB Hallmark database specifically showed the involvement of downregulated DEGs in EMT, hypoxia, and other major tumorigenic pathways. The Mammalian Phenotype (MP) Ontology database showed that the knockdown of FGFRL1 might result in decreased tumor growth and size.

**Fig. 4:**
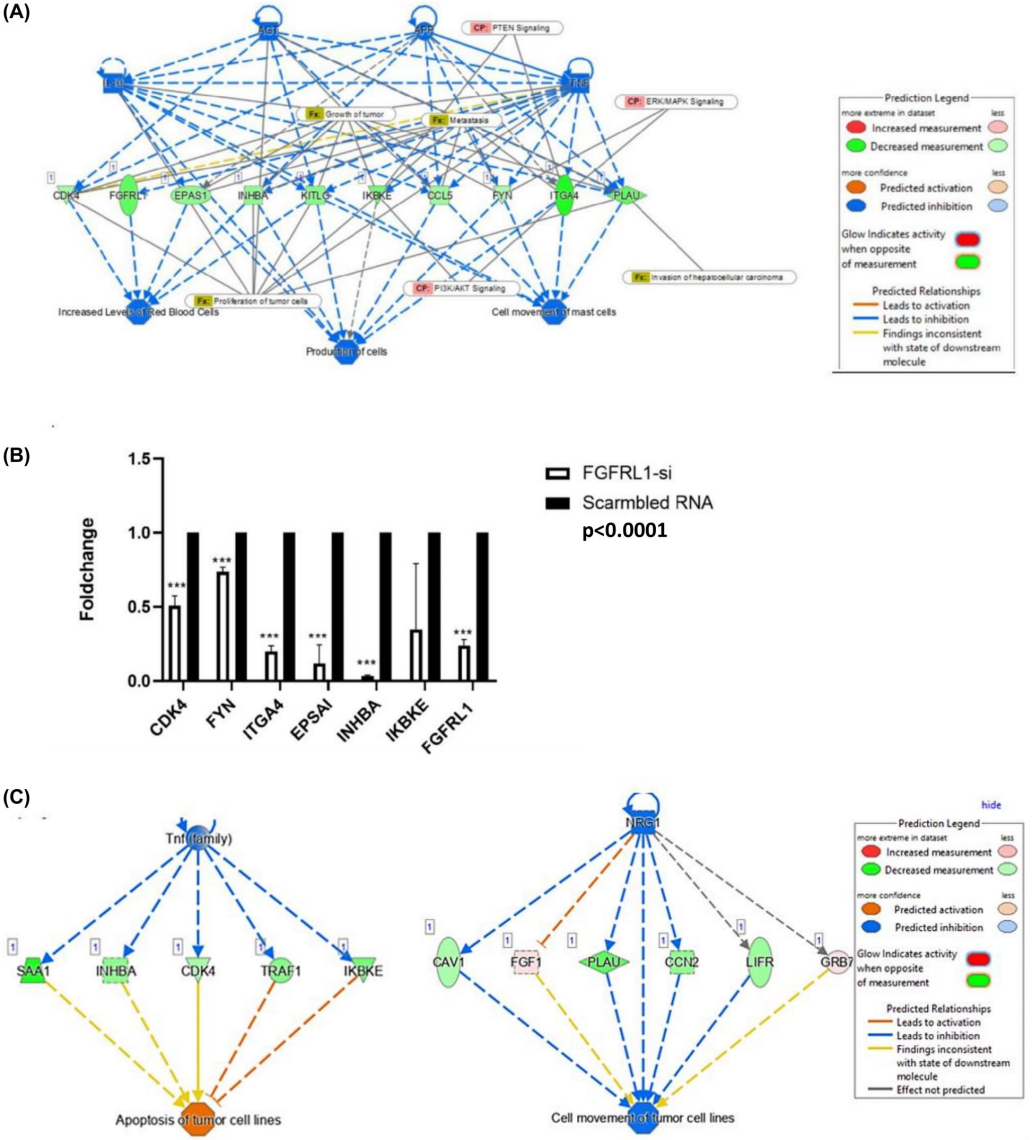
Biological pathway analysis of major clusters using IPA, Qiagen. (A) Predicted network analysis of DEGs using IPA. Each node represents a downregulated gene. The gray lines indicate pathways involving these genes, (B) qRT‐PCR quantified expression of total FGFRL1, and DEGs of the selected network in the esophageal cancer cells 48 h post‐FGFRL1 silencer transfection(Student's *t*‐test, *n* = 3 and values are presented as mean ± SD). (C) Downstream prediction network of downregulated cluster genes. **** indicates *P* < 0.0001, and values are presented as mean ± SD.

Next, the PPI network analysis of the DEGs and their functional pathway behavior was explored using Ingenuity Pathway Analysis software (IPA, QIAGEN). Wherein significantly downregulated genes and control‐specific genes were processed, and all clusters were scored according to vertex weighting, complex prediction, and optionally postprocessing to filter. The highest‐scoring cluster was selected as shown in Fig. 4A. The genes in this cluster were related to PI3K Akt signalling and epithelial‐mesenchymal transition (EMT) events.

First, we validated this differential expression of cluster genes from post‐FGFRL1 knockdown KYSE410 cells and observed a significant reduction in the mRNA expression of CDK4, IKBKE, FYN, EPSAI, ITGA4, and INHBA(*P* < 0.001) (Fig. 4B). Further, these genes were analyzed for downstream prediction networks, which predicted that their downregulated expression can trigger apoptosis of tumor cells and decrease cell movement ability (Fig. 4C).

**Fig. 5:**
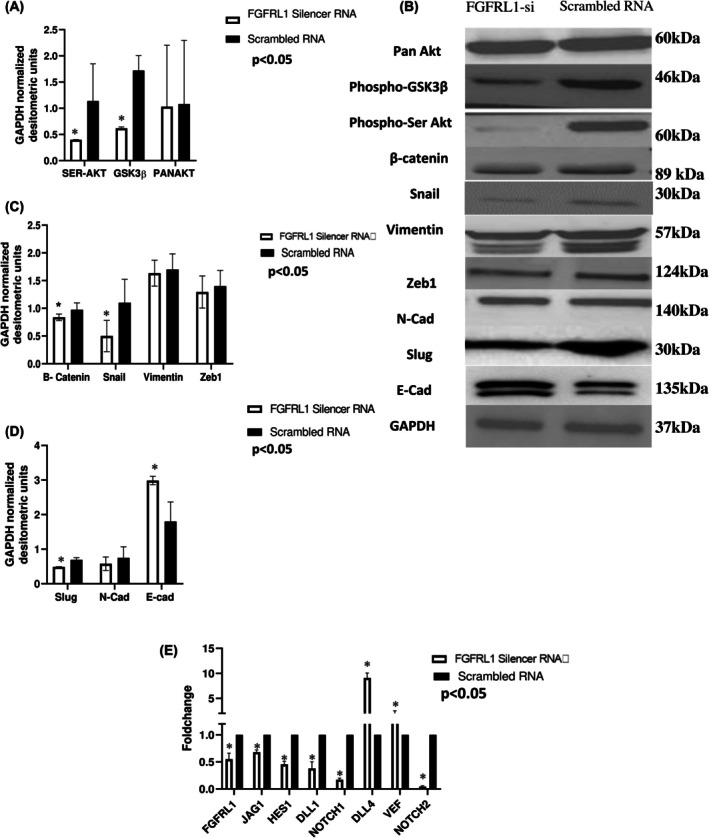
FGFRL1 expression modulation affects the PI3K‐Akt pathway and the expression of EMT markers. (A)–(D) Western blot experiments showed the expression levels of p‐SerAkt, phospho‐GSK3β, and pan‐Akt, along with EMT markers (Vimentin, β‐catenin, N‐cadherin, E‐cadherin, Slug, Snail, and Zeb1) post‐FGFRL1 downregulation(Student's *t*‐test, *n* = 3). (E) The expression of total FGFRL1, Notch ligands JAG1, DLL1, NOTCH1, NOTCH2, and the transcription factor HES1 was quantified by qRT‐PCR in esophageal cancer cells 48 h post‐FGFRL1 knockdown(Student's *t*‐test, *n* = 3). * indicates *P* < 0.05, and values are presented as mean ± SD.

As, the cluster genes were found to be involved in PI3K‐Akt signalling, which plays a major role in EMT, metastasis, and invasion, we explored the effects of FGFRL1 knockdown *in vitro* on downstream signalling pathways like PI3K‐Akt, Notch pathway, and EMT process. We found that the knockdown of FGFRL1 significantly reduced Phospho‐Akt and Phospho‐GSK3β expression. Furthermore, the expression of mesenchymal markers such as Snail, N‐cadherin, Vimentin, Zeb1, β‐catenin, and Slug protein levels was reduced whereas the expression of the epithelial marker E‐cadherin was significantly increased as compared to control post‐48 h transfection (*P* < 0.05) (Fig. 5A–D).

Also, the decreased expression of FGFRL1 in KYSE410 cells was found to be associated with the inactivation of Notch signalling, with a reduction in the expression of Notch ligands JAG1, DLL1, DLL4, and receptors NOTCH1 and NOTCH2, as well as the target gene HES1 as quantified by qRT PCR (*P* < 0.05) (Fig. 5E).

**Fig. 6:**
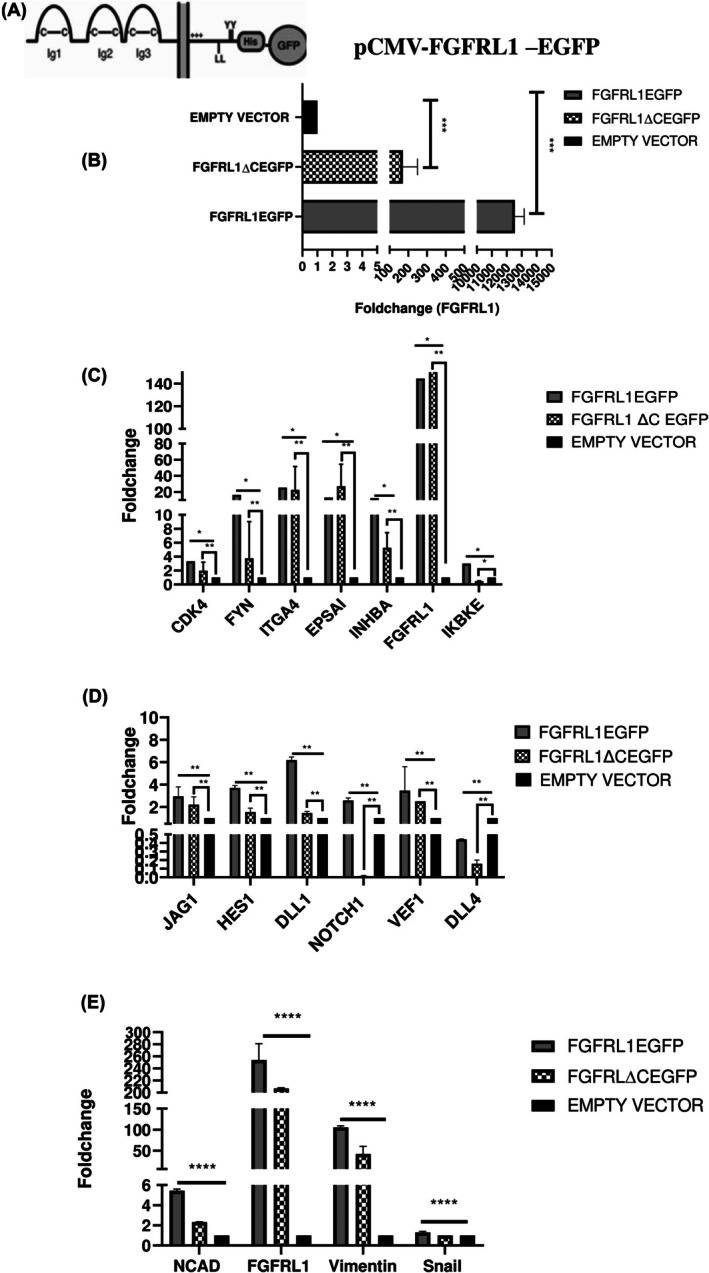
(A) Diagrammatic representation of pCMV‐FGFRL1‐EGFP. (B) mRNA expression level of FGFRL1 in the KYSE410, 48 post–FGFRL1–EGFP transfection. (C) Expression of total FGFRL1 and DEGs of the Selected Network was quantified by qRT‐PCR in the esophageal cancer cells 48 h post‐FGFRL1/FGFRL1ΔC‐transfection. (D) Expression of total FGFRL1, Notch ligands; JAG1, DLL1, NOTCH1, NOTCH2, DLL4, VEF and transcription factors HES1 was quantified by qRT‐PCR in the esophageal cancer cells 48 h post‐FGFRL1/FGFRL1ΔC transfection. (E) Expression of FGFRL1, AND EMT MARKERS (N‐CADHERIN, Vimentin, Snail) was quantified by qRT‐PCR in the esophageal cancer cells 48 h post‐FGFRL1/FGFRL1ΔC‐transfection. * indicates *P* < 0.05, ** indicates *P* < 0.01, *** indicates *P* < 0.001, **** indicates *P* < 0.0001, and Student's t‐test, *n* = 3, Values are presented as mean ± SD.

To complement these results, we overexpressed full‐length FGFRL1 as pCMV‐FGFRL1‐EGFP in KYSE410 cells (Fig. 6A). qRT‐PCR analysis showed that FGFRL1 overexpression remarkably increased FGFRL1 levels in KYSE410 (Fig. 6B) (*P* < 0.05). As expected, the expression of major cluster genes (CDK4, IKBKE, FYN, EPSAI, ITGA4, and INHBA) as well as the mRNA levels of EMT markers including N‐ Cad, Vimentin, and Snail (*P* < 0.05) and Notch signalling markers, were found to be upregulated post‐FGFRL1 overexpression (Fig. 6C–E) (*P* < 0.05).

To further delineate the region in the intracellular domain of FGFRL1 that might be involved in downstream signalling, we constructed the FGFRL1 C‐domain‐deleted construct, FGFRL1ΔC (450–504 aā). Interestingly, cells transfected with the FGFRL1ΔC construct showed longer retention of FGFRL1 protein as compared to its wild‐type counterpart (Fig. S1). Herein, we noticed that in FGFRL1ΔC construct transfected cells, only IKBKE shows reduced mRNA levels among all other cluster genes (*P* < 0.05). Whereas in the notch signalling pathway, only NOTCH1 and DLL4 seem to get affected post‐FGFRL1 C‐domain deletion (Fig. 6B–E) (*P* < 0.05). The EMT markers such as N‐cad, Vimentin, and Snail were also downregulated post‐FGFRL1ΔC (*P* < 0.001).

These results suggest that the downregulation of FGFRL1 is likely responsible for the constitutive decrease of major signalling pathways like the PI3K‐Akt pathway, Notch signalling, and the EMT process.

### FGFRL1 knockdown suppresses the migration and invasion potential of ESCC cells

**Fig. 7:**
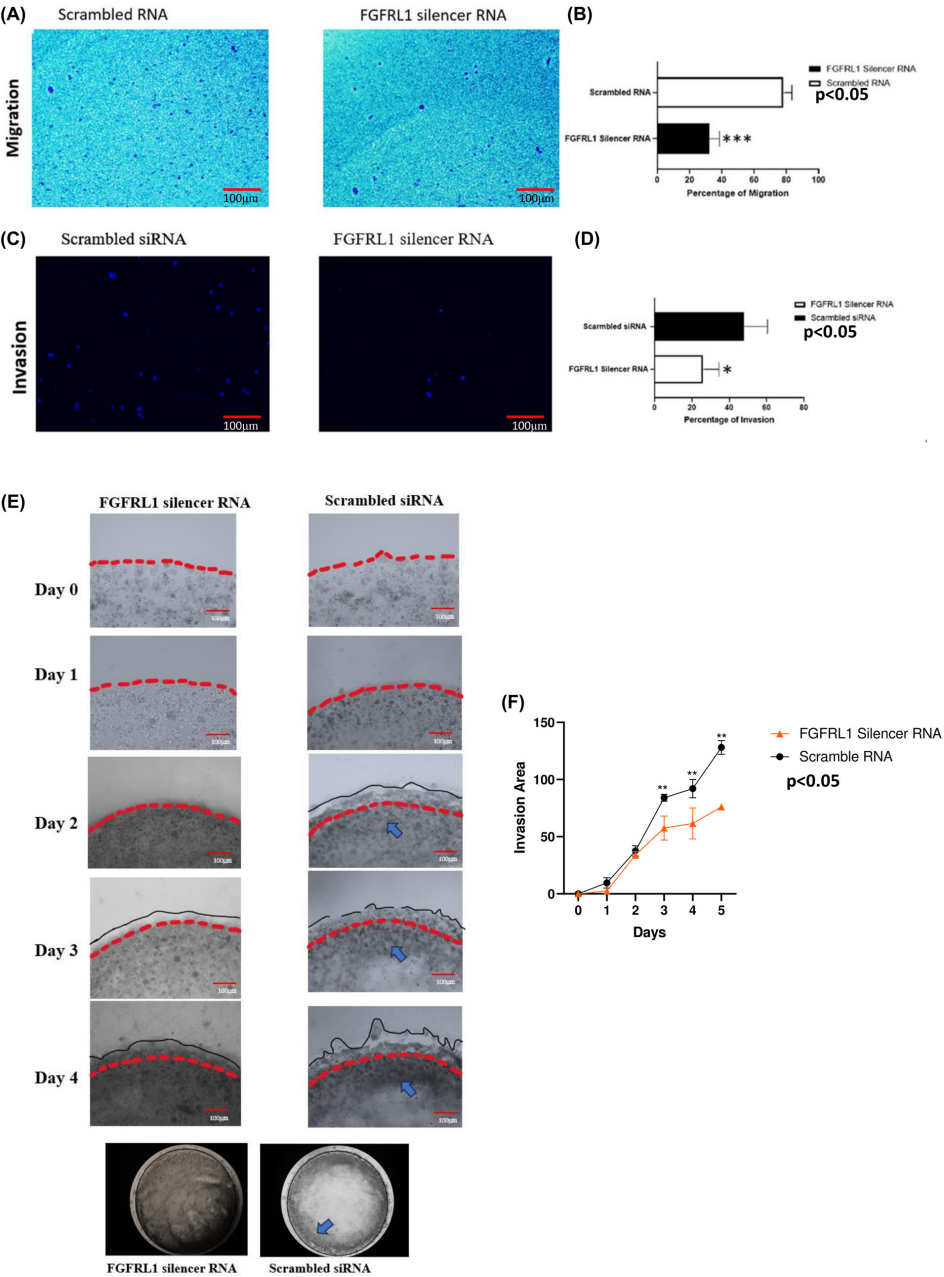
(A, B) Boyden chamber cell migration assay: The migration of FGFRL1 siRNA‐treated cells was lower as compared to scrambled siRNA‐treated cells (Student's *t*‐test, *n* = 3, ***P < 0.001), (C, D) Matrigel invasion assay: Significantly lower invasion capacity was observed in FGFRL1siRNA treated EC cells as compared to control (*P < 0.05). Size bar = 100 μm. (E, F) 3D drop Invasion assay: area invasion by migrating cells decreases post‐FGFRL1 knockdown as compared to control (Student's *t*‐test, *n* = 3, *P < 0.05).

We further evaluated the effect of FGFRL1 knockdown on the migration of KYSE‐410 cells using the Boyden Chamber assay. FGFRL1 siRNA resulted in significantly decreased migratory ability of KYSE‐410 cells at 48 h post‐FGFRL1 siRNA transfection (*P* < 0.001). There was a significantly reduced number of KYSE‐410 cells that had migrated through the chamber as compared to control, that is, 32 ± 9 and 78 ± 7 in the FGFRL1 si‐treated and scrambled RNA‐treated group, respectively (Fig. 7A–D). As the breakdown of the extracellular matrix is a critical determinant of metastasis, invasion is more closely linked to the metastasis process. Hence, a modified Boyden chamber coated with Matrigel was used, and the number of KYSE‐410 cells was 25 ± 8 and 54 ± 12 in the FGFRL1 knockdown group as compared to the negative control group, respectively (Fig. 7C,D). For further validation of this effect on invasive potential, we performed a 3D droplet assay wherein after 24 h of transfection, 50 000 cells/drop were plated with the Matrigel/ECM Gel. The results were captured from Day 0 to Day 6. At each time point, the area covered by total invading drop and the area of noninvasive, propagating cells is calculated. We observed that the area of invasion by migrating cells is 64% more in Control as compared to post‐FGFRL1 knockdown on Day 6. This indicates that FGFRL1 plays an important role in enhancing the invasive potential of EC cells (Fig. 7E,F). Complementary results were obtained post‐FGFRL1 overexpression. The area of invasion by cells on the 6th day is 56.2% more as compared to control, whereas FGFRL1ΔC overexpressed cells showed 38% less area of invasion as compared to wildtype (Fig. S2).

### FGFRL1 knockdown has a significant effect on the proliferation and clonogenic potential of esophageal carcinoma cell

**Fig. 8:**
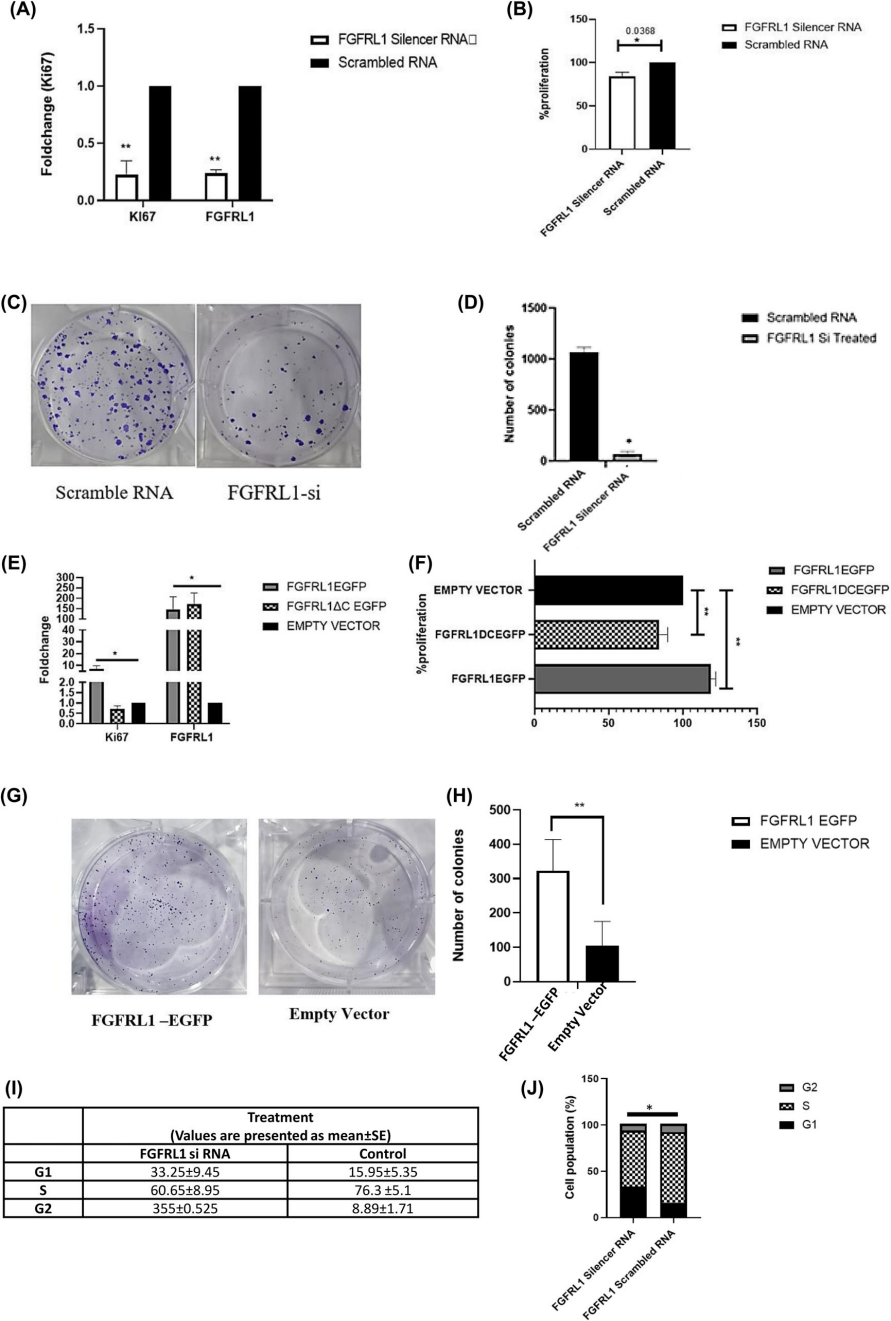
(A) Expression of total FGFRL1 and proliferation marker Ki67 was quantified by qRT‐PCR in the esophageal cancer cells 48 h post‐FGFRL1siRNA‐transfection. (B) Histogram representing the percentage of cell proliferation after transfection of FGFRL1 siRNA at 48 h in the KYSE‐410 cells. Effect of FGFRL1 knockdown on the colony formation potential of EC cells. (C) Representative images of colony formation assay after transfection of FGFRL1 siRNA or scrambled RNA for 7 days. (D) Histogram showing relative colony formation efficiency in the FGFRL1 siRNA or scrambled RNA group when normalized to the untreated. (E) Expression of total FGFRL1 and proliferation marker Ki67 was quantified by qRT‐PCR in the esophageal cancer cells 48 h post‐FGFRL1/FGFRL1ΔC vs Control transfection. (F) Histogram representing the percentage of cell proliferation after transfection of FGFRL1/FGFRL1ΔC vs Control at 48 h in the KYSE‐410 cells. Effect of FGFRL1 knockdown on the colony formation potential of ESCC cells. (G) Representative images of colony formation assay after transfection of FGFRL1 or Empty Vector for 7 days. (H) Histogram showing relative colony formation efficiency in the FGFRL1 or Empty Vector group when normalized to the untreated. (I) Cell cycle assay of control KYSE410 cells and FGFRL1 siRNA‐treated KYSE410 cells, (J) Stacked bar graph illustrates cell cycle distribution (*P* < 0.05) * indicates *P* < 0.05, ** indicates *P* < 0.01, Student's *t*‐test, *n* = 3, values are presented as mean ± SD.

To detect the effect of FGFRL1 gene knockdown and overexpression on cell growth, a WST assay was carried out in the FGFRL1 knockdown group, negative control group, overexpression group, and control group. The data were measured at 48 h post‐transfection. We also evaluated the levels of proliferation marker Ki67 and the change in percentage proliferation post‐FGFRL1 knockdown and overexpression. The levels of Ki67 were reduced by 60% post‐FGFRL1 knockdown, indicating that FGFRL1 may affect proliferation (Fig. 8A). We also observed a reduction in proliferation percentage post‐FGFRL1 knockdown as measured by cytotoxicity assay. The proliferation rate of the FGFRL1 knockdown group was found to be reduced by 28.7% post‐FGFRL1 knockdown as compared to the negative control group (*P* < 0.001) (Fig. 8B). Moreover, a significantly reduced clonogenic potential of up to 65% in KYSE‐410 cells after FGFRL1 knockdown was observed (*P* < 0.01) (Fig. 8C,D). Similar results were obtained in another ESCC cell line, KYSE‐30 cells, where FGFRL1 knockdown significantly reduced proliferation at 48 h post‐transfection (*P* < 0.01). The wound healing assay showed markedly decreased migration at both 24 and 48 h (*P* < 0.05). Moreover, clonogenic potential also reduced compared with controls (*P* < 0.05) (Fig. S4).

As expected, the levels of proliferation marker Ki67 and also the change in percentage proliferation were found to be increased post‐FGFRL1 overexpression (Fig. 8E–H). We also overexpressed the FGFRL1ΔC construct, wherein we noticed no change in proliferation percentage post‐FGFRL1 C‐domain deletion (Fig. 8E–H) (*P* < 0.05). DNA content analysis by flow cytometry showed a significant increase in the percentage of KYSE‐410 cells in the G1 phase from 15.95 ± 5.35% to 33.25 ± 9.45%, at 48 h post‐FGFRL1si transfection as compared to the control (*P* = 0.016) and a substantial decrease in S phase, from 76.3 ± 5.1% to 60.65 ± 8.95% (Fig. 8I,J).

### Correlation of FGFRL1 with EMT‐related markers in esophageal carcinoma

**Fig. 9:**
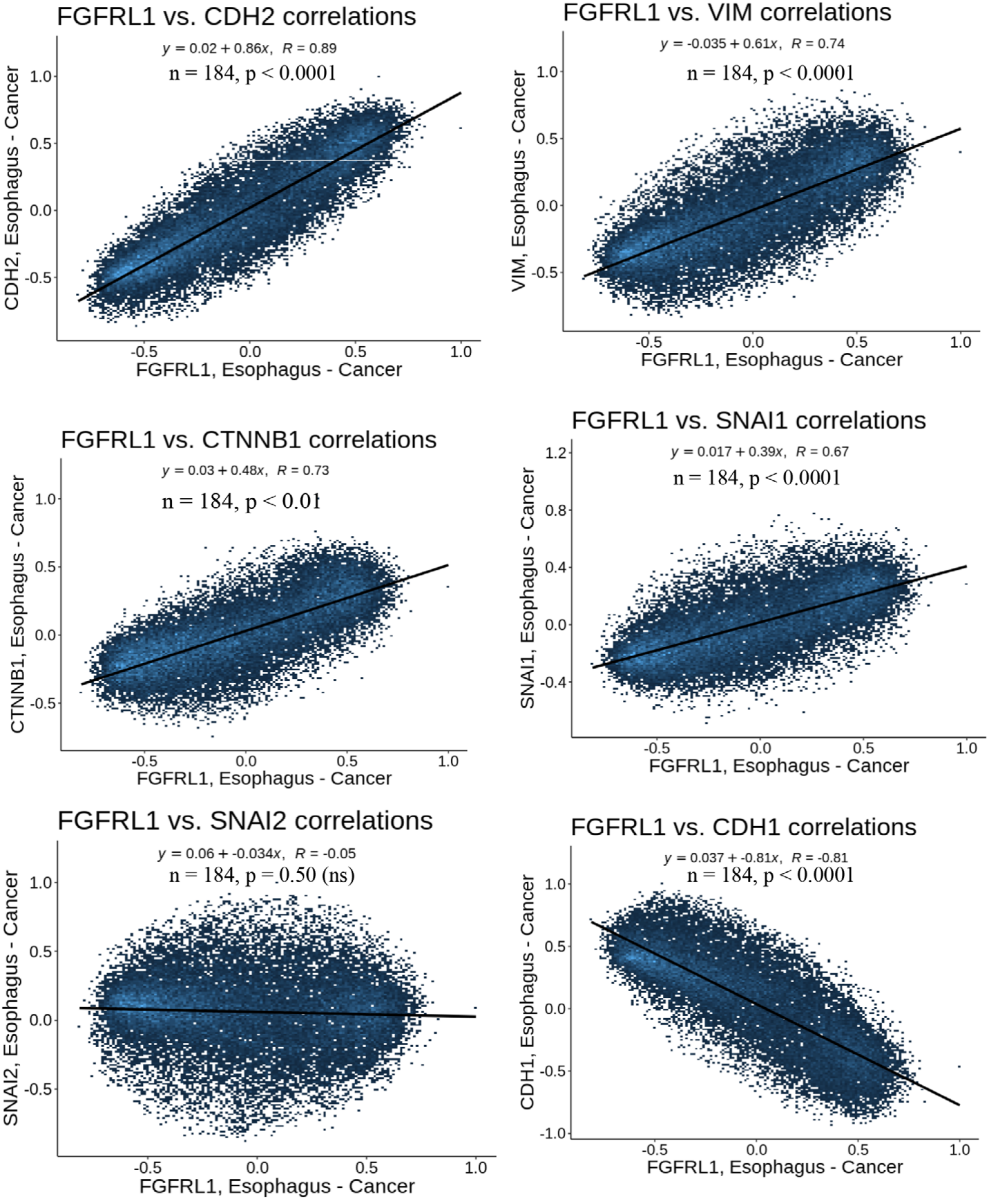
Correlation of EMT markers with FGFRL1 in the TGCA dataset of esophageal carcinoma patients using Correlation analysis.

In EC, as we observed, FGFRL1 promotes the invasion and metastasis of cells and plays a role in the development of tumors. Next, we used bioinformatics to analyze the database samples, hoping to find the EMT‐related genes and their relation with FGFRL1 upregulation in EC. The genes involved in the EMT process were significantly positively correlated with FGFRL1 in EC. The established EMT markers including mesenchymal markers such as CDH2(N‐CADHERIN), Vimentin, β‐catenin, SLUG, and Snail genes positively correlated with FGFRL1, whereas the epithelial marker such as CDH1, (E‐CADHERIN) showed a negative correlation (Fig. 9). These results further supported that FGFRL1 may be playing a crucial role in affecting cell invasion by upregulation of mesenchymal markers in the EMT process.

## Discussion

Deregulation of FGFR signalling plays a critical role in carcinogenesis. However, the clinical and functional significance of FGFRL1, the fifth member of this family, remains largely unexplored. Until now, only a limited number of studies have been carried out to investigate its expression in cancer patients. Recently, Yu *et al*. 2022 reported increased protein expression of FGFRL1 in prostate cancer patients and demonstrated its association with prognosis [11]. Increased FGFRL1 mRNA expression has also been studied in oral squamous cell carcinoma (OSCC) and ovarian cancer (OC) tissues [12, 13]. However, the expression of FGFRL1 in EC has not yet been investigated. Thus, in the present study, we performed immunohistochemical analysis of FGFRL1 in EC patients. Our study revealed a significant upregulation of FGFRL1 protein expression in EC tissues as compared to adjacent nonmalignant tissues (*P* < 0.001, AUC = 0.817). Both the histological subtypes EAC and ESCC showed upregulation of FGFRL1 in 92.8% and 90.2% of tissues, respectively. Interestingly, 72.5% of preneoplastic specimens showed significant FGFRL1 upregulation with an AUC of 0.822, suggesting dysregulation of FGFRL1 expression in the early stages of EC development. These results point toward its potential diagnostic value, which may further be used to differentiate EC from the normal subset. Interestingly, we found that smokers exhibited higher FGFRL1 expression compared to nonsmokers, an important observation given that tobacco consumption is a major risk factor for EC in the Indian population. Furthermore, our clinical findings, though based on a modest sample size, provide a strong rationale for validation in larger, independent cohorts.

Although initially thought to be a decoy receptor, recent accumulating evidence points towards the possibility of active signalling downstream of FGFRL1. To investigate the role of FGFRL1, we used RNAi to knock down FGFRL1 in the ESCC cell line (KYSE410), followed by NGS postknockdown to identify DEGs and associated pathways. Pathway analysis of DEGs using the Enricher tool revealed several pathways converging on EMT. Among the most significantly altered pathways were the Notch signalling pathway, class I PI3K signalling events, SMAD pathway, N‐cadherin signalling events, and epithelial‐to‐mesenchymal transition. To further confirm whether these major pathways are affected post‐FGFRL1 knockdown, we first examined the levels of PI3K‐Akt pathway proteins (phospho‐AKT, phospho‐GSK3β). We observed that FGFRL1 knockdown affects the activation of PI3K‐Akt pathway proteins. FGFRL1 downregulation reduces phospho‐AKT and phospho‐GSK3β levels, which may lead to GSK3β‐mediated phosphorylation of Snail and subsequent upregulation of E‐cadherin. Additionally, we observed a decrease in SLUG levels, another transcriptional regulator of E‐cadherin, alongside Snail [14]. Next, we examined the Notch signalling pathway, another enriched pathway in our analysis that is highly upregulated in cancers. This pathway involves the interaction of DLL and JAG ligands with Notch receptors, leading to proteolytic cleavage and the release of the Notch intracellular domain (NICD). NICD interacts with corepressor protein complexes, removing them from the promoter regions of target genes such as HES1, cMyc, and Nde, thereby increasing their expression [15]. We observed a reduction in mRNA levels of the target gene HES1 and ligands such as NOTCH1, NOTCH2, DLL1, DLL4, and JAG1 post‐FGFRL1 knockdown, and complementary results were seen after overexpression.

Both these major pathways have been reported to contribute to EMT progression in cancer. To validate our findings, we next studied the expression of EMT markers post‐FGFRL1 knockdown in ESCC cells. We observed significant dysregulation in the levels of major EMT markers (E‐cadherin, Snail, SLUG, ZEB1, β‐catenin, N‐cadherin, Vimentin) post‐FGFRL1 knockdown. Hence, we recorded decreased protein levels of mesenchymal markers (Snail, SLUG, ZEB1, β‐catenin, N‐cadherin, Vimentin) and increased expression of the epithelial marker E‐cadherin. Since EMT occurs when the expression of E‐cadherin (CDH1), which maintains epithelial characteristics, is suppressed, and the expression of N‐cadherin (CDH2), which promotes mesenchymal characteristics, is increased [16]. This profile change looks like a shift toward epithelial transitioning due to FGFRL1 knockdown, which might inhibit invasive and migratory properties in cells. This was also observed in both our 2D and 3D invasion experiments, which indicated FGFRL1 knockdown significantly reduced the migration and invasion potential of EC cells, while FGFRL1 overexpression enhanced these properties. We also observed a positive correlation between FGFRL1 and EMT markers in EC patient data from the TCGA dataset using bioinformatic tools. *In vitro* cell function assays showed that FGFRL1 knockdown results in significantly decreased migration and invasion ability of EC cells. Also, FGFRL1 knockdown led to significantly reduced expression of the proliferation marker Ki67 as well as a decrease in percentage proliferation post‐FGFRL1 knockdown. Our flow cytometry findings also suggest that silencing FGFRL1 leads to an accumulation of cells in the G1 phase, indicating a G1 phase arrest. These findings point toward an oncogenic role of FGFRL1 in EC. While our data provide strong evidence for the clinical and functional significance of FGFRL1 in EC, one of the limitations of the present study is that functional experiments were conducted using the esophageal squamous cell carcinoma cells (KYSE410/KYSE30). Given that our clinical data show significance of FGFRL1 in both ESCC and EAC, functional validation in EAC‐derived cell lines is warranted.

Simultaneously, we also analyzed differentially expressed genes using the Ingenuity Pathway Analysis (IPA) tool for cluster analysis of NGS post‐FGFRL1 knockdown. Out of 236 significantly downregulated genes and 51 control‐specific genes, these six highest‐scoring cluster genes (IKBKE, FYN, CDK4, ITGA4, INHBA, and EPSAI) were found to be involved in EMT, PI3K/Akt signalling, and the SMAD pathway. The mRNA expression of these cluster genes was significantly reduced post‐FGFRL1 knockdown. Conversely, overexpression of wild‐type FGFRL1(pCMV‐FGFRL1‐EGFP) induced a significant increase in the mRNA expression of these cluster genes. The first cluster gene identified is FYN, an Src kinase, as a significant DEG. FYN is required for enhanced invasion and migration [17]. We observed reduced levels of FYN post‐FGFRL1 knockdown, indicating that modulation of FGFRL1 expression affects FYN mRNA levels. Another hub gene identified is Integrin subunit alpha 4 (ITGA4), which is known to upregulate and support the nuclear localization of GLI1 protein, resulting in the activation of Hedgehog signalling [18]. Interestingly, FGFRL1 knockdown has previously been reported to result in diminished levels of GLI1 and GLI2, leading to downregulation of the Hedgehog (Hh) signalling pathway in ovarian carcinoma cells [9]. Since we noted reduced ITGA4 expression in post‐FGFRL1 knockdown, it might lead to inactivation of Hedgehog signalling. Next, the cluster gene EPSAI or HIF2α, a subunit of the HIF unit, was also found to be reduced post‐FGFRL1 knockdown, and FGFRL1 overexpression complemented these results. EPSAI or HIF2α is known to be involved in angiogenesis, the cell cycle, and cell metabolism [19, 20]. Among the CDKs, CDK4, one of our cluster genes, is a well‐known cyclin D‐dependent driver of the cell cycle, division, and survival [21]. In our cell function assays, we observed that FGFRL1 overexpression increases cell survival and clonogenic potential in EC cells, potentially via increased CDK4 expression. Another cluster gene, INHBA (Inhibin), is known to interact with TGF‐β signalling, which further activates EMT through ZEB1, SLUG, and Snail [22], corroborating our results where reduced protein levels of EMT markers were observed post‐FGFRL1 knockdown. The downregulation of these key cluster genes—IKBKE, ITGA4, CDK4, FYN, INHBA, EPSAI—post‐FGFRL1 knockdown, and their upregulation postoverexpression, underscores the critical role of FGFRL1 in pathways regulating tumorigenesis, metastasis, and cell cycle regulation. These findings emphasize the significance of these genes as potential targets of FGFRL1‐regulated downstream pathways.

Another interesting aspect of FGFRL1 that distinguishes it from other FGFRs is the lack of a tyrosine kinase domain. FGFRL1 features a 100‐bp‐long C‐terminal domain that contains conserved motifs, including a dileucine peptide, a tandem tyrosine‐based motif (YXXΦ), and a histidine‐rich sequence [4, 5, 12]. Initially believed to be a decoy receptor, emerging evidence suggests its downstream signalling is linked to various diseases, including cancer. Also, Zhuang et al. (2010) in a genomic study demonstrated that certain motifs within this unique C‐terminal domain of FGFRL1 resemble those found in 40 different surface receptors, including ITAM/ITIM, putative Src homology domain‐2 (SH2)‐binding motifs, and PDZ‐interacting motifs, as observed in Nectin receptors [6]. In this way, FGFRL1 might be able to signal information via these predicted motifs, from the outside of the cell to the interior, even though it lacks the tyrosine kinase domain typically associated with the classical FGFRs. This hypothesis was then mechanistically proven by Silva et al. (2013), wherein they demonstrated that FGFRL1 interacts with SHP‐1 phosphatase via its SH2‐binding motif at the C‐terminal in insulin secretory granules [7].

Interestingly, in the present study, we observed that the C‐terminal domain‐deleted FGFRL1 (FGFRL1ΔC) mutant exhibited greater retention at the plasma membrane compared to wild‐type FGFRL1 (Fig. S1). This observation aligns with a previous report by Silva *et al*. (2013), where the authors noted enhanced plasma membrane localization of wild‐type FGFRL1 with C‐terminal truncation, potentially due to decreased recycling or endocytosis of the receptor [7]. Additionally, we observed that FGFRL1ΔC overexpression led to decreased levels of the Notch1 receptor in EC cells, further supporting the involvement of FGFRL1‐mediated signalling in its regulation. One possible explanation may be that FGFRL1 expression increases GSK3β phosphorylation (as shown by us in the present study), leading to its degradation and subsequent upregulation of Snail, which is known to regulate NOTCH1 expression [23]. Thus, the C‐terminal domain of FGFRL1 may be involved in downstream signalling that regulates NOTCH1 expression via Snail. Further, the FGFRL1ΔC mutant led to significantly reduced levels of IKBKE, NOTCH1, and DLL4 compared to wild‐type FGFRL1 overexpression, suggesting that the C‐terminal domain may play a role in modulating their expression as well. As mentioned earlier, FGFRL1 affects pathways such as MAPK and ERK, which might activate or repress transcription factors (e.g., NF‐κB, STATs) that in turn might regulate IKBKE expression. Further, studies may be done to evaluate the involvement of the C‐terminal domain of FGFRL1 with potential adaptor/downstream proteins. Thus, we conclude that FGFRL1 is significantly upregulated in EC and preneoplastic tissues. FGFRL1 knockdown disrupts EMT via PI3K‐Akt and Notch signalling, reducing migration, invasion, and proliferation. The C‐terminal domain of FGFRL1 may play a crucial role in its downstream signalling. These findings suggest FGFRL1 may be a key player in EC progression and could be further explored as a potential diagnostic marker in esophageal carcinoma.

## Conclusion

Our findings underscore the oncogenic role of FGFRL1 in esophageal carcinoma, where it facilitates cellular proliferation, invasion, and metastatic potential. The data suggest that FGFRL1 may potentiate EMT and invasive behavior through modulation of the PI3K‐Akt‐Notch signalling axis. These results provide a foundation for considering FGFRL1 as a potential diagnostic marker and therapeutic target. However, further research and *in vivo* validation are warranted to delineate the precise molecular mechanisms driving FGFRL1‐mediated tumorigenesis in EC.

## Conflict of interest

The author(s) declare no conflict of interest concerning the content of this paper. The authors declare that they have no known competing financial interests or personal relationships that could have appeared to influence the work reported in this paper.

## Author contributions

All authors contributed to the study conception and design. Investigation and data curation were performed by AS^1^ and conceptualization, investigation, and supervision were done by RS. The first draft of the manuscript was written by AS^1^ and all authors commented on previous versions of the manuscript. AS^2^ and DG are the clinicians who provided the tissue samples and carried out endoscopic procedures for the same. All authors (AS^1^, RS, AS^2^, DG) read and approved the final manuscript.

## Supporting information


**Fig. S1.** C‐domain deletion increases membrane retention of FGFRL1 in esophageal cancer cells.
**Fig. S2.** FGFRL1 promotes cell invasion in 3D drop assay, attenuated by C‐domain deletion.
**Fig. S3.** FGFRL1 shows membrane and cytoplasmic expression in EC tissues.
**Fig. S4.** FGFRL1 knockdown has a significant effect on the proliferation and clonogenic potential of ESCC cell line KYSE30.
**Fig. S5.** Basal expression and knockdown validation of FGFRL1 in esophageal cancer cell lines.
**Table S1.** Primer sequences used for gene expression analysis.

## Data Availability

The datasets generated and/or analyzed during the present study are available in the Dryad repository https://doi.org/10.5061/dryad.9p8cz8wvs.
