# Supplementary material for: FGFR Like1 drives esophageal cancer progression via EMT, PI3K/Akt, and notch signalling: insights from clinical data and next‐generation sequencing analysis
Source: FEBS Open Bio. 2026 Jan 2;16(5):979–99. doi: 10.1002/2211-5463.70167 (PMC13145353; doi:10.1002/2211-5463.70167)
Supplement: Supplementary file 1 — Fig. S1. C‐domain deletion increases membrane retention of FGFRL1 in esophageal cancer cells. Fig. S2. FGFRL1 promotes cell invasion in 3D drop assay, attenuated by C‐domain deletion. Fig. S3. FGFRL1 shows membrane and cytoplasmic expression in EC tissues. Fig. S4. FGFRL1 knockdown has a significant effect on the proliferation and clonogenic potential of ESCC cell line KYSE30. Fig. S5. Basal expression and knockdown validation of FGFRL1 in esophageal cancer cell lines. Table S1. Primer sequences used for gene expression analysis. [file FEB4-16-979-s001.docx]

**Supplementary Data**

FGFR Like1 drives esophageal cancer progression via EMT, PI3K/Akt, and notch signaling: insights from clinical data and NGS analysis

Aprajita Srivastava^1^, Dr. Anoop Saraya^2^, Dr. Deepak Gunjan^2^, Dr. Rinu Sharma^1,*^

1. **University School of Biotechnology, Guru Gobind Singh Indraprastha University,**

**Dwarka, New Delhi, India.**

**2. Department of Gastroenterology, All India Institute of Medical Science,**

**Ansari Nagar, New Delhi, India**

***Corresponding author at the University School of Biotechnology,**

**Guru Gobind Singh Indraprastha University,**

**Sector - 16C, Dwarka, New Delhi -110078, India.**

**Contact:+91 9899011197**

**E-mail address:** [**rinu.sharma@ipu.ac.in**](mailto:rinu.sharma@ipu.ac.in) **[Dr. Rinu Sharma]**

**Supplementary Figure 1. : C-domain deletion increases membrane retention of FGFRL1 in esophageal cancer cells**

**Supplementary Figure 1: C-Domain deletion of FGFRL1 expression was associated with increased retention of FGFRL1 on the membrane as compared to wild-type full-length FGFRL1. (Scale bar, 20x-50μm and 40x-20 μm).**

**Supplementary Figure 2. FGFRL1 promotes cell invasion in 3D drop assay, attenuated by C-domain deletion**

A)

B)

**Supplementary Figure 2: (A) Representative phase-contrast images showing 3D invasion of cells overexpressing FGFRL1–EGFP, FGFRL1ΔC–EGFP, or Empty Vector at Day 0, Day 2, Day 3, Day 4, and Day 6.
(B) Quantification of invaded area measured over time. FGFRL1 overexpression significantly enhances cell invasion compared to the Empty Vector control (Student’s *t*-test, *n* = 3 biologically independent experiments, *p* < 0.05), whereas deletion of the C-terminal domain reduces invasion relative to wild-type FGFRL1 (Student’s *t*-test, *n* = 3 biologically independent experiments, ***p* < 0.001). Data represent mean ± SD.**

**Supplementary Table 1: Primer sequences used for gene expression analysis are listed.**

| S.No. | Gene Name | Forward Primer | Reverse Primer |
| --- | --- | --- | --- |
| 1 | **Vimentin** | **TGTCCAAATCGATGTGGATGTTTC** | **TTGTACCATTCTTCTGCCTCCTG** |
| 2 | **VWF** | **GCTGACACCAGAAAAGTGCC** | **GTCCCCAATGGACTCACAGG** |
| 3 | **CDH2 (N-Cad)** | **TTACAGTGCAGTCTTATCGAAGG** | **CAACTTCTGCTGACTCCTTCAC** |
| 4 | **Snail** | **CGGAAGCCTAACTACAGCGA** | **CAGATGAGCATTGGCAGCGA** |
| 5 | **Ki-67** | **CGGATTATACCTGGCCTTCC** | **GTCACATTCAATACCCCTTCCA** |
| 6 | **NOTCH-1** | **TACAAGTGCGACTGTGACCC** | **ATACACGTGCCCTGGTTCAG** |
| 7 | **NOTCH-2** | **CAACCGCCAGTGTGTTCAAG** | **GAGCCATGCTTACGCTTTCG** |
| 8 | **DLL-4** | **CATCATCGAAGCTTGGGAGC** | **CACATAGTGGCCGAAGTGGT** |
| 9 | **JAG-1** | **GATCGCCTGCTCAAAGGTCT** | **GACTGGAAGACCGACACTCG** |
| 10 | **DLL-1** | **GGAGAAAGTGTGCAACCCTG** | **TGCAGTAGTTCAGGTCCTGG** |
| 11 | **HES-1** | **GGAAGCTGAGCAAGGCAAAA** | **GCGGTTTACTTGCTTGGGT** |
| 12 | **FGFRL1** | **ACACAGCCCTCCAAGATGAG** | **GCAGGTTCTTCAGGCTCAGT** |
| 13 | **5s** | **GTCTACGGCCATACCACCCTG** | **AAAGCCTACAGCACCCGGTAT** |
| 14 | **EPAS1** | **AGCCTTGGAGGGTTTCAT** | **GAGGAGGGCAGTTGTTGTAG** |
| 15 | **CDK4** | **TTGCATCGTTCACCGAGATC** | **CTGGTAGCTGTAGATTCTGGCCA** |
| 16 | **IKBKE** | **TGTCACTGGGGCTGCAGAG** | **GTCGAAGCCCCAGCACTTG** |
| 17 | **INHBA** | **AAGTCGGGGAGAACGGGTATGTGG** | **TCTTCCTGGCTGTTCCTGACTCG** |
| 18 | **ITGA4** | **TTCCAGAGCCAAATCCAAGAGTAA** | **AAGCCAGCCTTCCACATAACAT** |
| 19 | **FYN** | **GCACGGACAGAAGATGAC** | **CCAATCACGGATAGAAAGT** |

**Supplementary Figure 3:**

**FGFRL1 shows membrane and cytoplasmic expression in esophageal cancer (EC) tissues**

We observed both cytoplasmic and membrane staining of FGFRL1 in premalignant and tumor tissues, with higher intensity of expression as compared to non-malignant areas.

A)

B)

100μm

50μm

100μm

50μm

C)

D)

100μm

50μm

100μm

50μm

**Supplementary Figure 3: A) Enlarged inlet of non-malignant tissue showing no detectable expression of FGFRL1, B) preneoplastic (dysplastic) tissue, C) moderately differentiated Esophageal Squamous Cell Carcinoma(ESCC) tissue, and E) adenocarcinoma tissue showing membrane and cytoplasmic expression of FGFRL1.Images captured at 10× (scale bar: 100 µm) and 20× (scale bar: 150 µm) magnifications**

**Supplementary Figure 4**

**FGFRL1 knockdown has a significant effect on the proliferation and clonogenic potential of ESCC cell line KYSE30**

To assess the effect of FGFRL1 gene knockdown on cell growth in KYSE-30 cells, a wound healing assay, WST assay, and colony formation assay were also performed. Compared with scrambled siRNA-transfected cells, cells transfected with FGFRL1-specific siRNA showed a significant decrease of 62% (p ≤ 0.01) in protein expression levels of FGFRL1 post-48 hrs(Figure 02a-b). A significant reduction in cell proliferation was also observed at 48 hrs post-transfection in the FGFRL1 knockdown group compared with the scrambled RNA and blank controls (p < 0.01) (Figure02 c). In the wound healing assay, FGFRL1 knockdown markedly reduced cell migration at both 24 hrs and 48 hrs compared with the scrambled RNA and blank groups (p < 0.05) (Figure 02 d-e). Moreover, FGFRL1 knockdown significantly decreased the clonogenic potential of KYSE-30 cells compared with the scrambled RNA control group (p < 0.05) (Figure02 f-g). These findings indicate that FGFRL1 plays a critical role in regulating proliferation and colony-forming ability in KYSE-30 cells as well.

**Supplementary Figure 4. : (a-b) Protein level expression of FGFRL1 in the KYSE30, 48hrs post FGFRL1 knockdown. Lane 1, cells transfected with FGFRL1 silencer post 48 hrs; Lane 2, cells transfected with Scrambled RNA post 48 hrs. c) Histogram showing the percentage of cell proliferation after FGFRL1 siRNA transfection at 48 hours in KYSE-30 cells. (d-e) Wound healing assay images showing cell migration at 0, 24, and 48 hours post-transfection with FGFRL1 siRNA or scrambled RNA in KYSE-30 cells. Quantification of wound closure percentage is represented in the histogram. f) Representative images of colony formation assay following transfection with FGFRL1 siRNA or scrambled RNA for 7 days. g) Histogram showing the number of colonies formed in FGFRL1 siRNA or scrambled RNA groups normalized to untreated control. (p<0.05). * indicates P < 0.05, and Student’s t-test, n=3, Values are presented as means ± SD.**

**Supplementary Figure 5**

**FGFRL1 Basal Expression**

Western blotting demonstrated that FGFRL1 protein expression was markedly higher in KYSE410 cells compared to KYSE30 cells (Figure 03a-b). Furthermore, qRT-PCR analysis revealed that FGFRL1 mRNA levels were highest in KYSE410, followed by KYSE140 and KYSE30, while comparatively lower expression was observed in HEK293T and KYSE150 cells (Figure03c). These findings show that FGFRL1 is differentially expressed across esophageal cancer cell lines with the strongest expression in KYSE410. Compared to the endogenous expression of FGFRL1 and scrambled RNA controls, siRNA-mediated knockdown of FGFRL1 significantly reduced its mRNA expression (*p < 0.05).

**Supplementary Figure 5 (a) Western blot analysis showing FGFRL1 expression in KYSE410 and KYSE30 cells with GAPDH as loading control. (b) Densitometric quantification of FGFRL1 protein expression normalized to GAPDH in KYSE30 and KYSE410 cells. Bars represent mean ± SD from two replicates. (c) Relative FGFRL1 mRNA expression levels in different cell lines (KYSE410, KYSE140, KYSE30, HEK293T, and KYSE150). d) Expression level of FGFRL1 post-knockdown as compared to control and blank. (p<0.05) , * indicates P < 0.05, and Student’s t-test, n=3, Values are presented as means ± SD.**
